# Factors to improve odds of success following medial opening-wedge high tibial osteotomy: a machine learning analysis

**DOI:** 10.1186/s12891-024-07441-x

**Published:** 2024-04-24

**Authors:** Hong Yeol Yang, Yong Gwan Shin, Hyun Ho Shin, Ji Hoon Choi, Jong Keun Seon

**Affiliations:** 1 Department of Orthopaedic Surgery, Chonnam National University Medical School and Hospital, 322, Seoyang-ro 322 Hwasun-gun, Chonnam, 58128 Republic of Korea; 2 R&D Center, XRAI inc, Gwangju, 61186 Republic of Korea

**Keywords:** Treatment success, High tibial osteotomy, Knee osteoarthritis, Machine learning, Prediction, Random forest

## Abstract

**Background:**

Although high tibial osteotomy (HTO) is an established treatment option for medial compartment osteoarthritis, predictive factors for HTO treatment success remain unclear. This study aimed to identify informative variables associated with HTO treatment success and to develop and internally validate machine learning algorithms to predict which patients will achieve HTO treatment success for medial compartmental osteoarthritis.

**Methods:**

This study retrospectively reviewed patients who underwent medial opening-wedge HTO (MOWHTO) at our center between March 2010 and December 2015. The primary outcomes were a lack of conversion to total knee arthroplasty (TKA) and achievement of a minimal clinically important difference of improvement in the Knee Injury and Osteoarthritis Outcome Score (KOOS) at a minimum of five years postoperatively. Recursive feature selection was used to identify the combination of variables from an initial pool of 25 features that optimized model performance. Five machine learning algorithms (XGBoost, multilayer perception, support vector machine, elastic-net penalized logistic regression, and random forest) were trained using five-fold cross-validation three times and applied to an independent test set of patients. The performance of the model was evaluated by the area under the receiver operating characteristic curve (AUC).

**Results:**

A total of 231 patients were included, and 200 patients (86.6%) achieved treatment success at the mean of 9 years of follow-up. A combination of seven variables optimized algorithm performance, and the following specific cutoffs increased the likelihood of MOWHTO treatment success: body mass index (BMI) ≤ 26.8 kg/m^2^, preoperative KOOS for pain ≤ 46.0, preoperative KOOS for quality of life ≤ 33.0, preoperative International Knee Documentation Committee score ≤ 42.0, preoperative Short-Form 36 questionnaire (SF-36) score > 42.25, three-month postoperative hip-knee-ankle angle > 1.0°, and three-month postoperative medial proximal tibial angle (MPTA) > 91.5° and ≤ 94.7°. The random forest model demonstrated the best performance (F1 score: 0.93; AUC: 0.81) and was transformed into an online application as an educational tool to demonstrate the capabilities of machine learning.

**Conclusions:**

The random forest machine learning algorithm best predicted MOWHTO treatment success. Patients with a lower BMI, poor clinical status, slight valgus overcorrection, and postoperative MPTA < 94.7 more frequently achieved a greater likelihood of treatment success.

**Level of evidence:**

Level III, retrospective cohort study.

## Background

Medial opening-wedge high tibial osteotomy (MOWHTO) is a reliable treatment option for medial compartment osteoarthritis of the knee with varus alignment in young patients [1]. The benefits of MOWHTO have been demonstrated in multiple studies showing satisfactory clinical results, slowed progression of knee osteoarthritis, and delayed conversion to total knee arthroplasty (TKA) [2–8].

Previous studies have shown various survival rates ranging from 74 to 92% after 10 years of follow-up [9–12]. Several potential risk factors for failed MOWHTO have been identified: older age, higher body mass index (BMI), progressed stage of osteoarthritis, and undercorrected alignment of the lower extremity appear to be related to poor outcomes or survival after HTO [11, 13–16]. However, patients usually have multiple simultaneous risk factors, and due to the complex interactions and relationships between these factors, the ability to accurately predict and quantify the probability of failure of MOWTHO is challenging.

Machine learning is an artificial intelligence (AI) application that can analyze complex big data and generate algorithms to predict an outcome [17]. There has been a growing need to understand machine learning in medicine, and the influence of machine learning in orthopedic surgery has also recently attracted considerable interest [18–23]. If prognostic factors can be predicted preoperatively, appropriate preventive measures to delay the requirement for surgery can be performed to achieve successful postoperative outcomes in high-risk patients. Furthermore, factors that can allow clinicians to make an accurate prognosis and provide patients with customized risk predictions for outcomes are crucial.

To date, no study has developed a machine learning model to estimate the survival rates or success rates of MOWHTO based on predictive factors. Therefore, this study aimed to identify informative variables associated with the success of MOWHTO and to develop and internally validate machine learning algorithms to predict which patients will achieve treatment success following MOWHTO for medial compartmental osteoarthritis. We examined several variables that have previously been identified as factors influencing the outcome of MOWHTO and developed a machine learning algorithm based on them. Our hypothesis was that machine learning analysis could accurately predict patients who will achieve treatment success after MOWHTO for the follow-up duration.

## Methods

### Data and patients

This study was approved by the institutional review board of our institution, and all patients provided written informed consent. We retrospectively identified all patients who underwent MOWHTO at our institution for knee osteoarthritis between March 2010 and December 2015. Patients who underwent MOWHTO for isolated medial compartment osteoarthritis (Kellgren–Lawrence grad ≥ II) and who had varus malalignment with intact cruciate ligaments were included.

The exclusion criteria were as follows: (1) prior open knee procedures; (2) associated ligamentous insufficiency (anterior or posterior cruciate ligament) requiring reconstruction; (3) knee range of motion < 120° and flexion contracture > 15°; (4) either patellofemoral or lateral compartment osteoarthritis (Kellgren–Lawrence grade ≥ II); (5) inflammatory arthritis or traumatic osteoarthritis; and (6) a follow-up period < 5 years.

Ultimately, 231 knees (231 patients) with a mean follow-up period of 9 years (range, 5.0–11.5 years) were enrolled in the study.

### Surgical techniques and postoperative management

The goal was to shift the weight-bearing line to the Fujisawa point and to create 3–5° of postoperative mechanical valgus [24]. All surgical procedures were performed by two experienced orthopedic surgeons. Arthroscopic examinations were performed regularly at the time of MOWHTO. Detected meniscal tears or articular cartilage injuries were treated with debridement or microfracture based on the surgeon’s judgment. Following arthroscopy, MOWHTO was performed in a biplanar fashion according to the technique developed by the Arbeitsgemeinschaft für Osteosynthesefragen International Knee Expert group using a locking plate [25].

### Primary outcomes and candidate variables

The primary outcomes were defined as the achievement of a minimal clinically important difference (MCID) in the Knee Injury and Osteoarthritis Outcome Score (KOOS) for pain and clinical survival at least five years after HTO [26]. The endpoint of survival was conversion to TKA. Moreover, we selected 25 variables that have already been demonstrated to be predictive factors in the recent literature and significantly impact HTO outcomes and survival rates [11, 13–16, 27]. These included sex, age, BMI, meniscal status, cartilage status using the International Cartilage Repair Society (ICRS) grade, existence of kissing lesions, additional cartilage repair procedures, patient-reported outcome measures (PROMs) (KOOS, International Knee Documentation Committee [IKDC] score, Short-Form 36 [SF-36] questionnaire, Tegner activity scale score), and radiographic evaluations (mechanical hip-knee-ankle [HKA] angle), posterior tibial slope, medial proximal tibial angle [MPTA], and Kellgren–Lawrence grading). All the outcomes were assessed by two independent investigators blinded to the surgical procedures and study aim.

### Traditional statistical analysis

We defined clinical success as a well-functioning MOWHTO with a KOOS for pain that exceeded the MCID after a minimum follow-up of five years without conversion to TKA. An independent *t*-test and Pearson’s Chi-square test were used for continuous and categorical variables, respectively. The odds ratio of each variable was calculated. Multivariate logistic regression analyses were performed to examine the effects of these variables on treatment success. Receiver operating characteristic (ROC) curves with area under the curve (AUC) analyses were performed to evaluate model sensitivity and specificity. All statistical analyses were performed using SPSS (version 25.0; SPSS, Chicago, IL, USA), and *p* < 0.05 was considered statistically significant.

### Algorithm development

A binary classifier model that predicts the possibility of treatment success based on 25 variables was developed and constructed using Python. An 80:20 random sample split was used to partition the study population into training (*n* = 184) and independent test (*n* = 47) sets for algorithm development and internal validation, respectively. Five-fold cross-validation with five iterations of training using the training set was used to develop five unique machine learning algorithms: extreme gradient boosting (XGBoost), multi-layer perception (MLP), support vector machine (SVC), random forest (RF), and elastic-net penalized logistic regression (ENPLR).

### Model performance assessment

We evaluated the performance of the models using independent test sets that were not used for model training and measured their performance using the Brier score and AUC. These metrics were used to identify the best-performing model. An ROC curve plots the probability of correctly identifying positive cases against the probability of falsely identifying positive cases at different threshold settings. The AUC score assigns a score of 1 to a perfect predictor and 0.5 to a predictor with random guesses. The Brier score is calculated by averaging the squared difference between the outcome and model prediction probabilities to provide an overall performance measure. Lower Brier scores indicate better model performance. We used the Brier score obtained from a traditional logistic regression as a null model, and models with better performance than this null model were considered adequate. Finally, the optimal model was selected based on the lowest Brier score.

### Model fidelity and application development

It is important to understand individual predictions made by classifiers. The explanation of individual predictions allows informed decision-making about how much predictions can be trusted and provides insight to improve the model. Ribeir et al. [28] introduced LIME, which explains individual predictions using locally interpretable surrogate models. In this study, LIME provides quantitative data and visual representations of patient-specific predictions, enabling determination of what specific combinations of factors support or contradict the prediction that a specific patient will achieve treatment success with MOWHTO. We developed a web application that provides individualized prediction through the R packages ‘lime’ by Pedersen et al. [29] and ‘shiny’ by Chang et al. [30] However, in their current form, these predictions represent a proof-of-concept for machine learning in orthopedics and should not be used until additional validation studies are performed.

## Results

A total of 231 patients were included, and 200 patients (86.6%) achieved treatment success at a mean of 9 years of follow-up. The demographic characteristics and clinical data are summarized in Table 1.

**Table 1:** Baseline Demographic Characteristics and Clinical Data*

	Value
Sex	
Male	181 (78.4%)
Female	50 (21.6%)
Age	56.4 ± 6.1
BMI	25.6 ± 3.0
Meniscal status	
Intact	144 (62.3)
<1/3 resected	75 (32.5)
>1/3 resected	12 (5.2)
Initial cartilage status†	
MFC	3.3 ± 0.7
MTP	3.0 ± 0.8
Kissing lesion	55 (23.8%)
Cartilage repair procedure	126 (54.5%)
Preoperative ROM	138.6 ± 9.1
Preoperative KOOS	
Pain	41.8 ± 6.5
Symptoms	50.3 ± 6.9
Activities of daily living	52.7 ± 5.6
Sports and recreation	25.2 ± 4.9
Quality of life	29.2 ± 5.7
Preoperative IKDC	39.2 ± 5.0
Preoperative SF-36 PCS	42.7 ± 6.3
Preoperative tegner activity scale score	2.0 ± 0.4
Preoperative Kellgren-Lawrence grade	2.7 ± 0.5
Preoperative HKA	6.8 ± 2.6
Preoperative MPTA	85.8 ± 2.8
Preoperative posterior tibial slope	7.4 ± 3.8
Postoperative 3months HKA	2.6 ± 2.6
Postoperative 3months MPTA	93.1 ± 2.8
Postoperative 3months posterior tibial slope	9.3 ± 3.4
Achievement of treatment success for MOWHTO	200 (86.5%)

*Values are presented as the mean ± standard deviations or n (%)

†Initial cartilage status was graded at the time of initial high tibial osteotomy according to the International Cartilage Repair Society grading system

BMI, body mass index; MFC, medial femoral condyle; MTP, medial tibial plateau; ROM, range of motion; KOOS Knee Injury and Osteoarthritis Outcome Score; IKDC International Knee Documentation Committee; SF-36 PCS Short Form-36 Physical Component Summary; HKA hip–knee–ankle; MPTA medial proximal tibial angle; MOWHTO, medial opening-wedge high tibial osteotomy

### Traditional analysis − logistic regression analysis

Based on the multivariate regression analysis, the preoperative KOOS for pain, preoperative SF-36 Physical Component Summary (PCS) score, and preoperative MPTA influenced treatment success after MOWHTO (Table 2). The AUC of this regression model was 0.66 (95% confidence interval (CI), 0.420–0.890).

**Table 2:** Multiple Logistic Regression Analysis for Predictors of MOWHTO Treatment Success

	Multivariate Analysis	
	Estimate (95% CI)	*P* Value*
Sex (female vs. male)	0.409 (− 0.870 to 1.851)	0.549
Age	−0.022 (− 0.106 to 0.059)	0.588
BMI	0.045 (− 0.118 to 0.228)	0.603
Meniscal status	−0.021 (− 0.937 to 0.945)	0.963
Initial cartilage status (MFC)	0.535 (− 0.313 to 1.363)	0.201
Initial cartilage status (MTP)	−0.624 (− 1.599 to 0.281)	0.186
Kissing lesion	−0.865 (− 2.478 to 0.729)	0.285
Cartilage repair procedure	−0.436 (− 1.603 to 0.662)	0.445
Preoperative ROM	−0.263 (− 0.710 to 0.036)	0.185
Preoperative KOOS		
Pain	−0.143 (− 0.259 to − 0.036)	**0.010**
Symptoms	0.072 (− 0.042 to 0.190)	0.217
Activities of daily living	−0.055 (− 0.183 to 0.070)	0.390
Sports and recreation	0.076 (− 0.038 to 0.191)	0.191
Quality of life	−0.035 (− 0.133 to 0.057)	0.458
Preoperative IKDC	0.118 (− 0.037 to 0.258)	0.109
Preoperative SF-36 PCS	0.092 (0.020 to 0.167)	**0.012**
Preoperative Tegner activity scale score	−0.177 (− 1.587 to 1.235)	0.803
Preoperative HKA	−0.116 (− 0.346 to 0.104)	0.305
Preoperative MPTA	−0.292 (− 0.517 to − 0.088)	**0.006**
Preoperative posterior tibial slope	−0.019 (− 0.147 to 0.113)	0.766
Postoperative 3months HKA	−0.174 (− 0.040 to 0.394)	0.110
Postoperative 3months MPTA	−0.067 (− 0.210 to 0.074)	0.352
Postoperative 3months posterior tibial slope	0.084 (− 0.139 to 0.313)	0.461

*Bold indicates a *P* value < 0.05 (statistically significant difference)

†Initial cartilage status was graded at the time of initial high tibial osteotomy according to the International Cartilage Repair Society grading system

BMI, body mass index; MFC, medial femoral condyle; MTP, medial tibial plateau; ROM, range of motion; KOOS Knee Injury and Osteoarthritis Outcome Score; IKDC International Knee Documentation Committee; SF-36 PCS Short Form-36 Physical Component Summary; HKA hip–knee–ankle; MPTA medial proximal tibial angle

### Assessment of algorithm performance

**Fig. 1:**
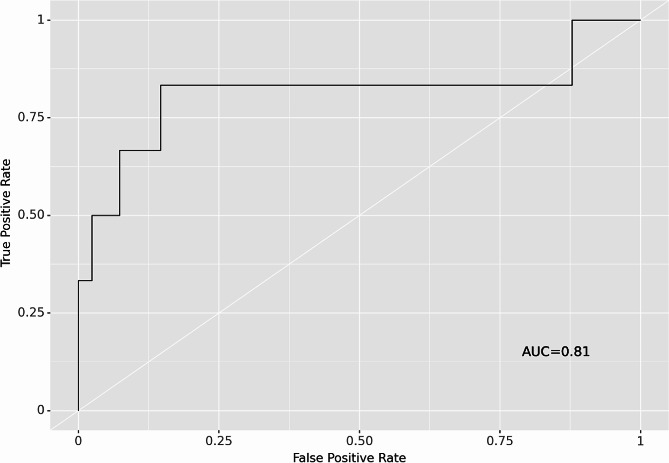
Receiver operating characteristic (ROC) curve for the random forest machine learning model. AUC = area under the ROC curve

The relative algorithm performances of the five algorithms are described in Table 3. The best-performing algorithm was the random forest model with an AUC of 0.81 (95% CI, 0.771–0.849) (Fig. 1). The Brier score was 0.09 (95% CI, 0.089–0.099). The null-model Brier score was 0.107 (95% CI 0.101–0.114), indicating that this algorithm appropriately calibrated predictions.

**Table 3:** Performance of Each Machine Learning Algorithm in the Independent Test Set of Patients*

	XGBoost	Multi-Layer Perception	Support Vector Machine	Elastic Net Logistic Regression	Random Forest
Accuracy	0.86 (0.84 to 0.87)	0.86 (0.85 to 0.87)	0.86 (0.86 to 0.87)	0.86 (0.86 to 0.87)	0.88 (0.87 to 0.89)
Sensitivity	0.16 (0.10 to 0.21)	0.0 (0.0 to 0.0)	0.0 (0.0 to 0.0)	0.11 (0.06 to 0.16)	0.17 (0.11 to 0.24)
Specificity	0.96 (0.95 to 0.98)	0.99 (0.99 to 1.0)	1.0 (1.0 to 1.0)	0.98 (0.97 to 0.99)	0.98 (0.98 to 0.99)
Precision	0.42 (0.27 to 0.58)	0.0 (0.0 to 0.0)	0.0 (0.0 to 0.0)	0.36 (0.21 to 0.51)	0.59 (0.41 to 0.77)
F1 Score	0.21 (0.14 to 0.28)	0.0 (0.0 to 0.0)	0.0 (0.0 to 0.0)	0.18 (0.10 to 0.26)	0.25 (0.17 to 0.33)
Brier Score	0.10 (0.09 to 0.11)	0.12 (0.11 to 0.12)	0.11 (0.11 to 0.11)	0.10 (0.09 to 0.11)	0.10 (0.09 to 0.10)
AUC	0.76 (0.73 to 0.80)	0.66 (0.60 to 0.73)	0.69 (0.64 to 0.74)	0.69 (0.63 to 0.75)	0.81 (0.77 to 0.85)

*Values are presented as means and 95% confidence intervals

### Importance of the features

**Fig. 2:**
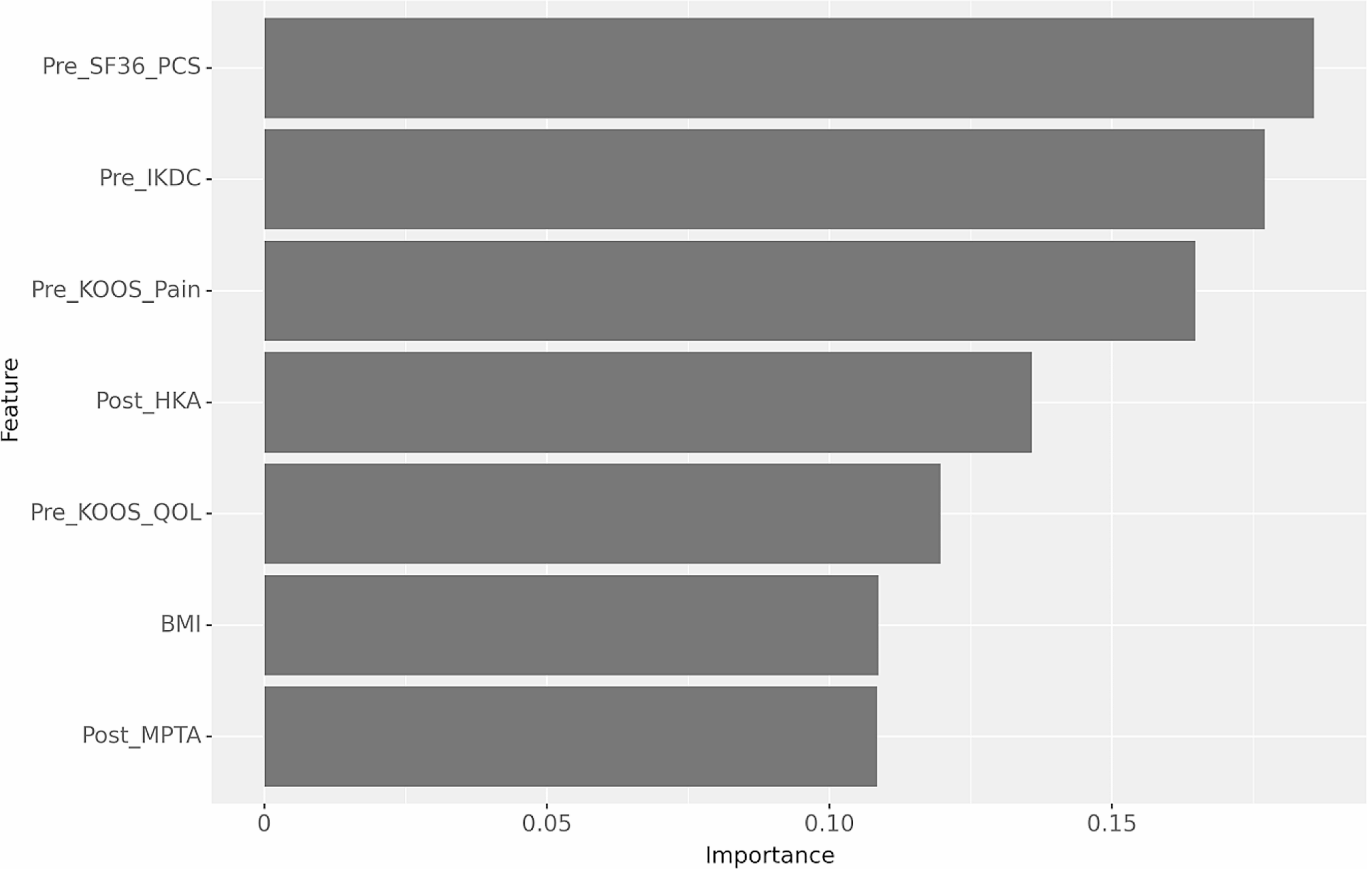
Feature importance plot for the random forest model based on the independent test set. Each predictive weight of each variable is compared among the other seven variables chosen from recursive feature elimination with cross-validation. KOOS = Knee Injury and Osteoarthritis Outcome Score, SF-36 PCS = Short-Form 36 questionnaire Physical Component Summary, IKDC = International Knee Documentation Committee, HKA = hip–knee–ankle, QOL = quality of life, BMI = body mass index, and MPTA = medial proximal tibial angle

We evaluated the importance ranks that indicated the importance of the input features for the random forest algorithm. The performance of the algorithm was optimized by combining seven variables: BMI, preoperative IKDC score, preoperative KOOS for pain, preoperative KOOS for quality of life (QOL), preoperative SF-36 PCS score, postoperative HKA angle, and postoperative MPTA (Fig. 2). We created 56 unique cases of LIME with 5,000 permutations to determine the relative contribution of these features to the overall predictions. This allowed us to determine the levels of each feature and ranges of values that either supported or contradicted treatment success for categorical and continuous variables. A BMI ≤ 26.8 kg/m^2^, preoperative IKDC score ≤ 42.0, preoperative KOOS for pain ≤ 46.0, preoperative KOOS for QOL ≤ 33.0, preoperative SF-36 PCS score > 42.25, postoperative HKA angle > 1.0°, and a postoperative MPTA > 91.5° and ≤ 94.7° were associated with treatment success for patients undergoing MOWHTO for medial compartmental osteoarthritis.

### Customized prediction application

**Fig. 3:**
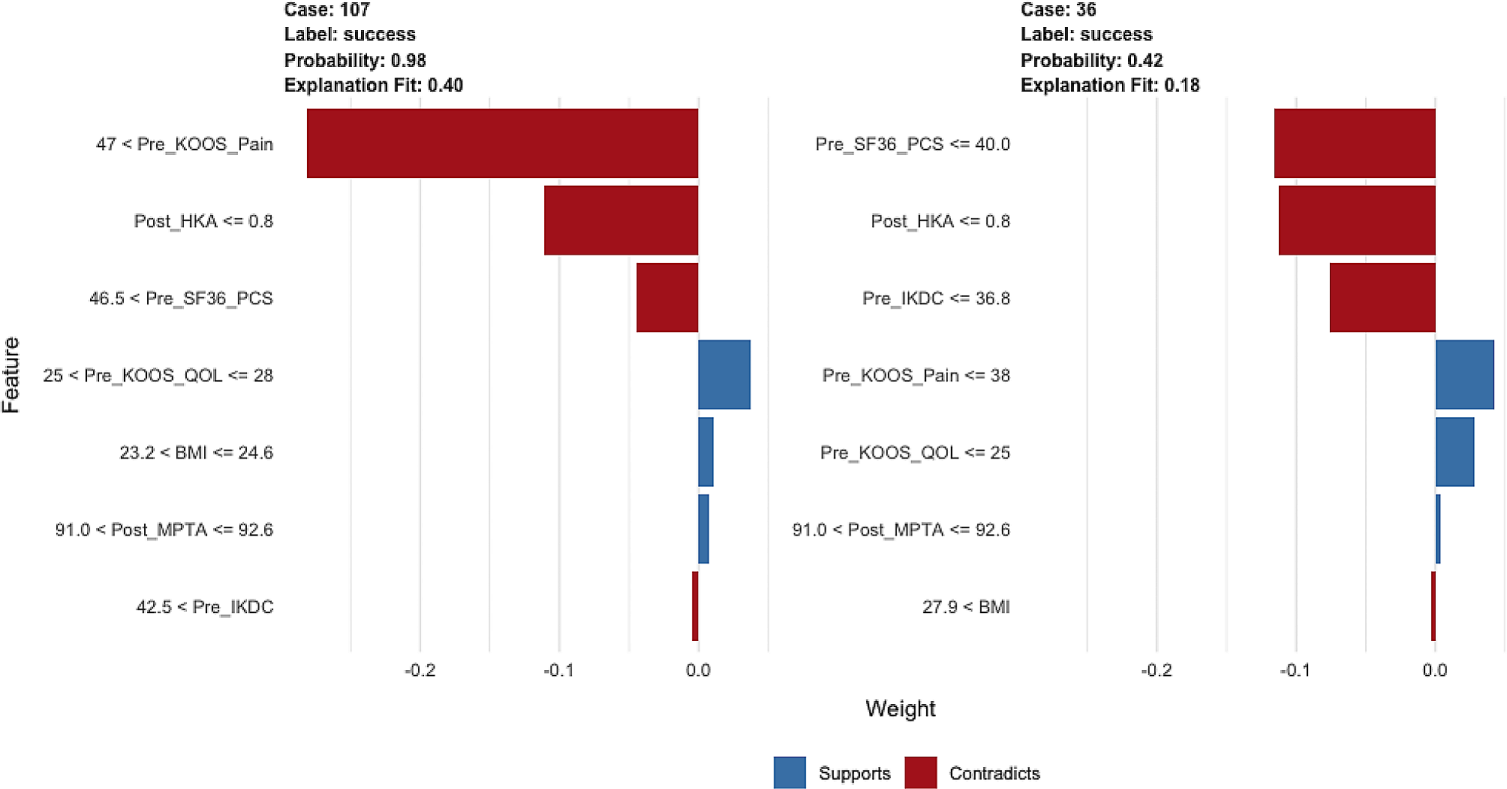
Demonstration of the possible clinical influence of the clinical decision-making tool derived from the random forest model. The probability of achieving treatment success following MOWHTO is 98.0% (left) and 42.0% (right). Factors marked in blue positively affected this patient’s ability to achieve treatment success. Factors marked in red had a negative impact. KOOS = Knee Injury and Osteoarthritis Outcome Score, SF-36 PCS = Short-Form 36 questionnaire Physical Component Summary, IKDC = International Knee Documentation Committee, HKA = hip–knee–ankle, QOL = quality of life, BMI = body mass index, MPTA = medial proximal tibial angle, and MOWHTO = medial opening-wedge high tibial osteotomy

We deployed the optimal algorithm as a web-based application (https://ailab.shinyapps.io/betterhto/). When the seven studied features are input into the algorithm, the probability of treatment success following MOWHTO is expressed as a percentage, and the importance of each feature used in the decision-making process is displayed in a graph (Fig. 3).

## Discussion

This study’s principal finding was that the random forest machine learning-based model demonstrated the best performance for predicting treatment success of MOWHTO at a mean of nine years postoperatively. The algorithm required only seven factors to predict treatment success: BMI, preoperative IKDC score, preoperative KOOS for pain, preoperative KOOS for QOL, preoperative SF-36 PCS score, postoperative HKA angle, and postoperative MPTA. The AUC to predict treatment success was 0.81, which exceeds the threshold for good performance of ≥ 0.8 [20], and this study enabled individualized prediction of treatment success after MOWHTO using a web-based system. Our results demonstrate that machine learning algorithms are promising new approaches in clinical situations wherein several variables must be comprehensively assessed, such as in predicting treatment success of MOWHTO in patients with medial compartmental osteoarthritis.

This is the first study to predict the patient-specific treatment success of MOWHTO by applying a machine learning model. Predicting treatment success of MOWHTO is challenging due to the heterogeneity and diversity of associated variables; discriminating factors must be identified to guide treatment decisions, and accurately quantifying this risk is difficult [15]. Our seven identified variables are simple and intuitive and can guide the patient-specific discussion regarding surgical options and realistic outcome goals.

Predictors of MOWHTO treatment success must be identified to reduce the risk of failure requiring TKA, and numerous predictors have been described to identify ideal candidates for MOWHTO [5, 11, 13–16, 31]. Bonasia et al. [11] identified positive (Ahlback grade 0 arthritis of the medial compartment and a good preoperative Knee Society score) and negative prognostic factors (age > 56 years and postoperative knee flexion < 120°) associated with MOWHTO outcomes in a case series of 140 patients. Jin et al. [16] analyzed the risk factors for survival after MOWHTO, and the main failure criteria were conversion to TKA and inferior PROMs. They presented a regression analysis showing that age ≥ 65 years, grade 4 cartilage damage in the medial compartment, and grade ≥ 2 cartilage damage in the lateral compartment negatively influenced outcomes after MOWHTO. Bouguennec et al. [13] demonstrated that survival factors reducing the risk of MOWHTO failure included female sex, age < 54 years, BMI < 25 kg/m^2^, Ahlback grade 1 or 2, varus articular component < 0.9°, HKA angle correction > 180°, and absence of a hinge fracture. Patients usually have multiple simultaneous risk factors, and some studies did not address the confounding effects of other variables, which should be controlled for accurate analysis of the true effects on postoperative outcomes.

Machine learning involves techniques that model complex relationships between variables to predict an outcome. Applications of predictive machine learning have broadly impacted the medical field, especially in orthopedic surgery, and facilitate surgeon decision-making [20–23, 32–38]. Batailler et al. [39] recently determined the main predictive factors for long-term HTO survival and proposed a predictive score that includes age, BMI, and the presence or absence of a joint line and is particularly useful in borderline cases for decision-making regarding potential HTO surgery. However, this is not a true prediction, and a machine learning model may be a helpful decision aid in daily practice to determine HTO indications. Martin et al. [21] performed a machine learning analysis of the Norwegian Knee Ligament Register (NKLR), identified important risk factors related to subsequent revision of primary anterior cruciate ligament (ACL) reconstruction, and developed a clinically meaningful calculator for predicting revision of primary ACL reconstruction. Kunze et al. [23] developed machine learning algorithms capable of providing patient-specific predictions of which athletes will achieve clinically relevant improvement in sports-specific function after hip arthroscopy for femoroacetabular impingement syndromes. Their machine learning algorithms demonstrated excellent performance in predicting achievement of an MCID in clinical scores. Using this framework, orthopedic surgeons may consider various treatment options preoperatively according to the patients’ individual risk profiles.

Notably, the random forest machine learning model identified seven variables that differed from those highlighted in traditional regression analyses as crucial for predicting MOWHTO treatment success. This discrepancy may be due to the distinct methodologies applied. Traditional regression highlighted only three parameters as significant in a multivariate analysis, emphasizing the reliance on statistical significance (P values) for variable selection. However, the random forest model employs a feature importance mechanism and ranks variables based on their contribution to model accuracy rather than based on statistical significance alone. This approach led to the identification of variables such as BMI, preoperative IKDC score, preoperative KOOS for pain, preoperative KOOS for QOL, preoperative SF-36 PCS score, postoperative HKA angle, and postoperative MPTA as important predictors. We identified two types of predictive factors: preoperative variables and postoperative variables at three months after the initial surgery. A low BMI and poor clinical status except for the preoperative SF-36 PCS score were important preoperative variables associated with MOWHTO treatment success. A BMI ≤ 26.8 kg/m^2^ was associated with MOWHTO treatment success using LIME analysis, which is in agreement with the findings of previous studies (25.0–27.5 kg/m^2^) [39, 40]. Overweight patients put excessive stress on the knee joint, which may accelerate degenerative changes and surgical outcomes. Bouguennec et al. [13] reported that a BMI < 25 kg/m^2^ was associated with reduced HTO failure, and Howells et al. [41] showed inferior PROMs at 5 years after HTO in patients with a BMI > 30 kg/m^2^. Patients with more inferior PROMs preoperatively showed a better prognosis than those with less inferior PROMs. A more severe clinical status for osteoarthritic knees may create an opportunity for improvement with MOWHTO. Preoperative features are dynamic and may be optimized following a trial of nonsurgical management. These findings are quite similar to those of previous reports in that significantly more patients with more severe disease before TKA are satisfied with their procedure than those with less severe degenerative changes [42, 43].

The ideal degree of correction has been extensively evaluated, and correction from neutral up to extreme valgus corrections is recommended [10, 44–46]. Our results showed that an HKA angle ≥ 1° was a positive factor for MOWHTO treatment success, which is consistent with the literature [13, 39], and undercorrection is generally associated with worse results [1, 47]. Thus, achieving adequate operative correction to a relevant angle is necessary for good long-term outcomes after MOWHTO [48, 49]. Furthermore, our findings suggest that unloading effects of MOWHTO led to clinical success, with no association with meniscal/chondral status or additional cartilage repair procedures and postoperative clinical outcomes.

Although the association between excessive joint line obliquity (JLO) and inferior outcomes after HTO has not yet been demonstrated, considering JLO for HTO is crucial [50–52]. In their biomechanical study, Nakayama et al. reported that an MPTA > 95° was unacceptable [53]. Schuster et al. [52] retrospectively reviewed 79 patients with medial knee osteoarthritis and demonstrated that an overcorrected MPTA (> 95°) was related to inferior clinical outcomes during long-term follow-up. Kim et al. [50] also assessed the influence of the MPTA on HTO outcomes at a minimum four-year follow-up using a propensity score matching analysis and suggested that although an excessively increased MPTA after HTO had no significant effects on clinical outcomes and cartilage deterioration in the lateral compartment, lateral compartment pain was experienced significantly more frequently. Thus, our findings of a postoperative MPTA ≤ 94.70° by LIME analysis are clinically relevant, and concerns about the potential side effects of a certain extent of overcorrection of MPTA should be understood cautiously for the orthopedic surgeons.

The random forest machine learning algorithm demonstrated excellent performance for predicting MOWHTO treatment success in patients with medial compartmental osteoarthritis compared with the conventional logistic regression model based on the AUC. The conventional logistic regression model is prone to overfitting of training data when used as a prediction model [54], often resulting in poorer performance when presented with new data, which makes it difficult to use clinically. The random forest model achieved a 12% relative Brier score reduction over the traditional logistic regression analysis. However, random forest classifiers may require more training data to produce robust results and may also contain unnecessary predictors; thus, further study of model optimization through feature selection of input variables and data augmentation is necessary. LIME was used to explain the individual predictions of our model. It applies to any predictive model and has no assumptions about the model. This is advantageous, especially when the model is trained to be noninterpretable. However, the results may vary at each execution because the data points are sampled without considering the correlation between variables. Alvarez-Melis et al. [55] reported that the explanations of two close points are very different from each other, indicating that there is instability in the explanation; thus, it sometimes can be difficult to trust.

Our study has certain limitations. First, this was a retrospective nonrandomized study, and relatively few patients were recruited. Further large-sample studies may improve the machine learning model performance. Second, although we considered a variety of machine learning methods, a model that was not considered might have had superior performance. Third, there are other possible predictors associated with MOWHTO treatment success that inherently vary depending on institutional protocols and surgeon preference that we could not evaluate in the present study. Fourth, for each of the five unique machine learning algorithms, we evaluated their performance using a training dataset through five-fold cross-validation with five iterations. However, developing a meta-algorithm, which was not used in the present study, would be better to explore the integration and improve the performance of the model. Fifth, the machine learning algorithms developed in this study were internally validated using an independent test set. However, external validation using data from other centers would strengthen the robustness of the algorithms. As this study recruited patients from a single center, observer or selection biases cannot be eliminated. Sixth, there might have been selection bias among the included patients. In Asian populations, MOWHTO tends to be more frequently performed in females than in males. Our findings might have been biased by the disproportionate female sex predominance [56–59]. Seventh, this study was based on a retrospective analysis performed at a single institution, which may restrict the generalizability of the results. Finally, it is possible that there are other important variables, including pre- to postoperative (delta) posterior tibial slope or contralateral ICRS grade, that could have been evaluated and may have led to alternative results. Furthermore, it could be more practical to include these variables with only one PROM because time-consuming PROM assessments are not routinely conducted in daily clinical practice. The addition of a slimmed web-based algorithm would allow the clinicians to reduce the number of input factors and thus facilitate obtaining information more quickly; however, it will likely have limited statistical significance. Further studies with larger sample sizes and additional crucial variables as well as collaborations with multiple centers are necessary to validate our findings.

## Conclusion

The correct indications are necessary to achieve MOWHTO treatment success. The random forest machine learning-based model used to evaluate patients who underwent MOWHTO showed demonstrated the best performance for predicting MOWHTO treatment success. According to our findings, patients with a lower BMI, poor clinical status, slight valgus overcorrection, and postoperative MPTA < 94.7° more frequently achieved a greater probability of treatment success. Our findings are clinically relevant and would allow patient and surgical information to guide shared clinical decision-making for patient-specific management.

## Data Availability

The datasets used and analyzed during the current study available are from the corresponding author upon reasonable request.

## Abbreviations

MOWHTO: Medial opening-wedge high tibial osteotomy
TKA: Total knee arthroplasty
BMI: Body mass index
AI: Artificial intelligence
MCID: Minimally clinically important difference
KOOS: Knee Injury and Osteoarthritis Outcome Score
PROMs: Patient-reported outcome measures
IKDC: International Knee Documentation Committee
SF-36: Short-Form 36
HKA: Hip-knee-ankle angle
MPTA: Medial proximal tibial angle
ROC: Receiver operating characteristic
AUC: Area under the curve
XGBoost: Extreme gradient boosting
MLP: Multi-layer perception
SVC: Support vector machine
RF: Random forest
ENPLR: Elastic-net penalized logistic regression
NKLR: Norwegian Knee Ligament Register
ACL: Anterior cruciate ligament
